# Identification of redox activators for continuous reactivation of glyoxal oxidase from *Trametes versicolor* in a two-enzyme reaction cascade

**DOI:** 10.1038/s41598-024-56429-z

**Published:** 2024-03-11

**Authors:** Saadet Alpdağtaş, Nina Jankowski, Vlada B. Urlacher, Katja Koschorreck

**Affiliations:** 1 Department of Biology, Van Yuzuncu Yil University, Van, 65080 Turkey; 2 Institute of Biochemistry, Heinrich Heine University Düsseldorf, Universitätsstraße 1, 40225 Düsseldorf, Germany

**Keywords:** Glyoxal oxidase, Copper radical oxidase, Redox activators, FDCA (2,5-furandicarboxylic acid), Biocatalysis, Oxidoreductases, Biocatalysis

## Abstract

Glyoxal oxidases, belonging to the group of copper radical oxidases (CROs), oxidize aldehydes to carboxylic acids, while reducing O_2_ to H_2_O_2_. Their activity on furan derivatives like 5-hydroxymethylfurfural (HMF) makes these enzymes promising biocatalysts for the environmentally friendly synthesis of the bioplastics precursor 2,5-furandicarboxylic acid (FDCA). However, glyoxal oxidases suffer from inactivation, which requires the identification of suitable redox activators for efficient substrate conversion. Furthermore, only a few glyoxal oxidases have been expressed and characterized so far. Here, we report on a new glyoxal oxidase from *Trametes versicolor* (*Tv*GLOX) that was expressed at high levels in *Pichia pastoris* (reclassified as *Komagataella phaffii*). *Tv*GLOX was found to catalyze the oxidation of aldehyde groups in glyoxylic acid, methyl glyoxal, HMF, 2,5-diformylfuran (DFF) and 5-formyl-2-furancarboxylic acid (FFCA), but barely accepted alcohol groups as in 5-hydroxymethyl-2-furancarboxylic acid (HMFCA), preventing formation of FDCA from HMF. Various redox activators were tested for *Tv*GLOX reactivation during catalyzed reactions. Among them, a combination of horseradish peroxidase and its substrate 2,2′-azino-di-(3-ethylbenzthiazoline sulfonic acid) (ABTS) most efficiently reactivated *Tv*GLOX. Through continuous reactivation of *Tv*GLOX in a two-enzyme system employing a recombinant *Moesziomyces antarcticus* aryl-alcohol oxidase (*Ma*AAO) almost complete conversion of 8 mM HMF to FDCA was achieved within 24 h.

## Introduction

**Figure 1:**
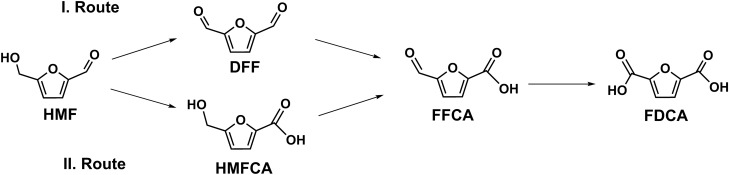
Reaction routes for HMF conversion into FDCA.

Due to climate change and fossil fuel deficiency, the demand for renewable and sustainable equivalents instead of fossil-derived commodities is increasing day by day^1^. In this context, waste plant biomass with its heterogenous composition has a promising potential for utilization in industrial biotechnology^2^. Lignocellulose accounts for the majority of this plant biomass and thus represents a readily available, sustainable, and renewable source for providing bio-based chemicals^3^. The environmental production of value-added commodities or building blocks from biomass is quite attractive and has gained increasing attention over the last years. For instance, the synthesis of renewable building blocks for polymeric materials like bioplastics has been intensively studied. FDCA (2,5-furandicarboxylic acid) is a promising precursor for the sustainable synthesis of bioplastics. FDCA can be derived from FFCA (5-formyl-2-furancarboxylic acid), that is produced from HMF (5-hydroxymethylfurfural) either over HMFCA (5-hydroxymethyl-2-furancarboxylic acid) or over DFF (2,5-diformylfuran) (Fig. 1). HMF is produced during the pretreatment of lignocellulosic biomass^4^. Several studies have dealt with the development of enzymatic routes for the environmentally friendly and sustainable production of FDCA from HMF^5–10^. Fungal enzymes involved in lignin degradation have been thoroughly investigated for this purpose^11–13^.

**Figure 2:**
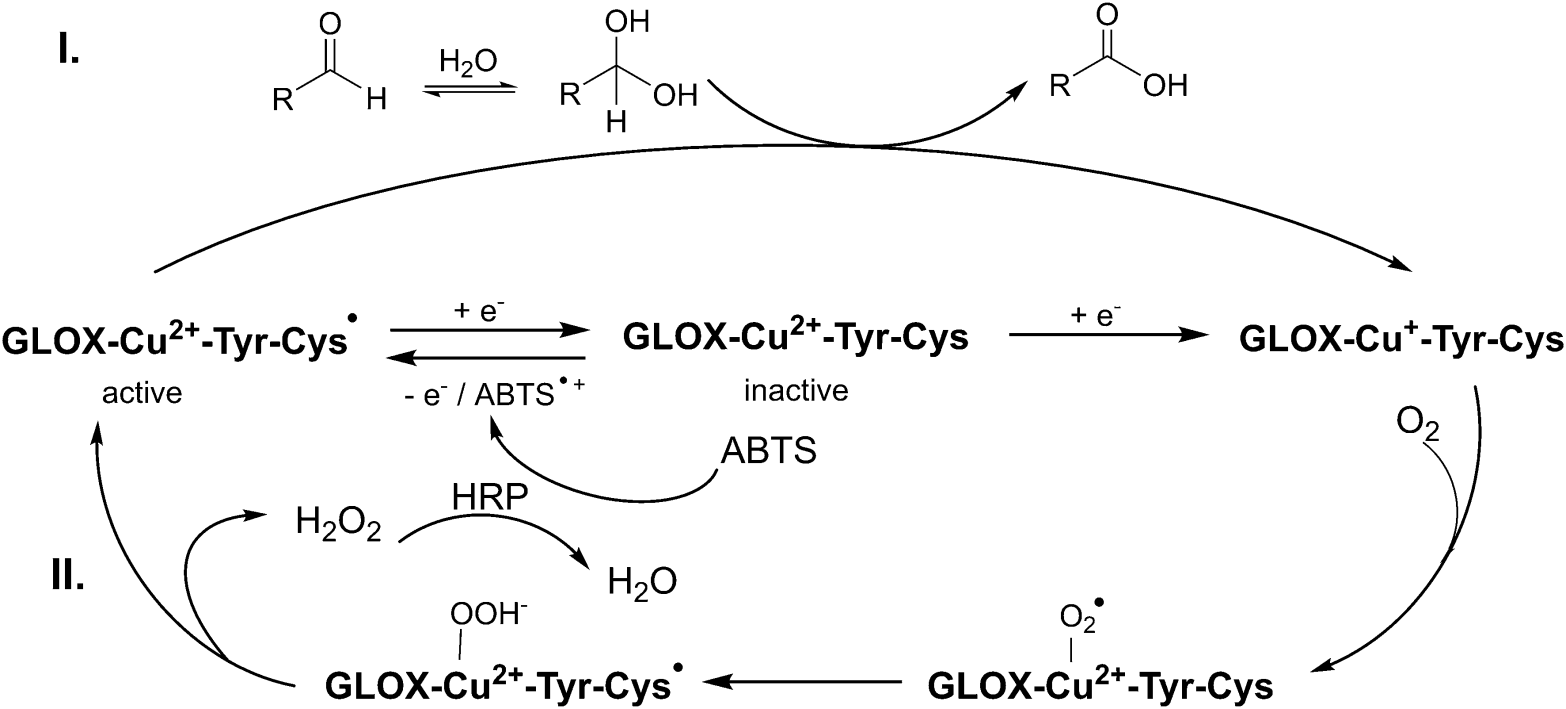
Reaction cycle of GLOX and reactivation by ABTS and horseradish peroxidase. I. oxidative half-reaction, II. reductive half-reaction.

Several organisms, particularly white-rot fungi, are efficient degraders of lignocellulose and the fungal secretome comprises a variety of enzymes like laccases, peroxidases, and H_2_O_2_-generating auxiliary enzymes, that contribute to lignin degradation^14^. Glyoxal oxidase (GLOX), discovered in secretomes of several fungi, is suggested to be involved in this process by fueling peroxide-depending ligninolytic peroxidases with H_2_O_2_. GLOX belongs to the family of copper radical oxidases (CROs) and catalyzes the reduction of O_2_ to H_2_O_2_ while oxidizing several aldehydes to carboxylic acids^15^. While other members of CROs like galactose oxidase (GalOx) have been extensively studied and some crystal structures of these enzymes are available, much less is known about GLOX enzymes. Similar to all CROs, these enzymes have a conserved active site with a mononuclear copper ion coordinated to an axial tyrosine, two histidines, and a cross-linked cysteine-tyrosyl radical cofactor^11,16^. Notably, GLOX as well as GalOx is inactivated during purification and requires initial activation by a strong oxidizing agent^17,18^. Inactivation is caused by the reduction of the active free-radical Cu(II)-state to the catalytically inactive non-radical Cu(I)-state (Fig. 2), as well as by high H_2_O_2_ concentrations. Activation of GLOX by regeneration of the oxidized radical form could be achieved by treating GLOX with high-redox potential inorganic oxidants like Na_2_IrCl_6_^18^ or other single oxidants generated by lignin peroxidase^19^. However, the activated enzyme is unstable with a half-life of 4 h for the radical form, and returns to the reduced inactive form^18^. Up to now, only GLOX from *Phanerochaete chrysosporium*^20,21^, from *Ustilago maydis*^22^, from *Myceliophthora thermophila*^3^, and three GLOX isoenzymes from *Pycnoporus cinnabarinus*^1,23^ were characterized. Nevertherless, the usefulness of GLOX for the production of value-added commodities from biomass has been demonstrated. For instance, GLOX was reported to oxidize glycerol and produce glyceraldehyde and glyceric acid^23,24^. In 2019, Daou et al. investigated the ability of three GLOX isoenzymes for the bioconversion of HMF to yield FDCA, when combined with an aryl-alcohol oxidase (AAO) to prevent HMFCA accumulation^1^. In this setup, 16% FDCA was formed from HMF along with 84% FFCA. However, further optimization of this promising two-enzyme approach for the complete conversion of HMF to FDCA is still required.

In the present study, we produced a new glyoxal oxidase from *Trametes versicolor* (*Tv*GLOX) in *Pichia pastoris* and investigated this enzyme for the production of FDCA from HMF in a two-enzyme reaction cascade. A suitable redox activator for continuous GLOX reactivation in the course of the reaction was identified and applied in our setup, giving almost complete conversion of 8 mM HMF to FDCA within 24 h.

## Results and discussion

### *Tv*GLOX production and enzyme purification

*Tv*GLOX was identified by protein BLAST search, using GLOX from *P. chrysosporium* (UniProtKB: Q01772.1) as a query. The *tvglox* encoding DNA sequence with the native signal sequence for secretion (NCBI accession number XM_008037054) was codon optimized using the online tool JCat^25^ and cloned into the pPICZA plasmid under the control of the *AOX1* promoter. The resulting plasmid was integrated into the *P. pastoris* X-33 genome by homologous recombination. Among 48 transformants, four *P. pastoris* colonies showing the most intensive green halo formation on methylglyoxal/HRP/ABTS-containing agar plates were selected for expression in shaking flasks. The transformant with the highest volumetric activity towards methylglyoxal of 0.23 U/ml was chosen for fed-batch cultivation in a 7.5 L bioreactor. After 9 days of fermentation, a volumetric activity of 19,000 U/l and a protein concentration of 1.6 g/l were obtained. Similar volumetric activity of 22.7 U/ml^21^ and protein concertation of 1–2 g/l^15^ were reported for expression of *P. chrysosporium* GLOX in *P. pastoris*.

*Tv*GLOX was concentrated from the supernatant and purified by hydrophobic interaction chromatography. The purified enzyme displayed a specific activity towards methylglyoxal of 4.0 U/mg. During purification, *Tv*GLOX changed its color from light green, attributed to the oxidized form of the enzyme, to purple color, which was observed for GLOX from *P. cinnabarinus* as well^23^.

Purified *Tv*GLOX had a molecular mass of around 80 kDa (Fig. S1), while the calculated molecular mass of native *Tv*GLOX is 58 kDa. Four potential *N*-glycosylation sites were found in *Tv*GLOX (Fig. S2). *N*-deglycosylation of *Tv*GLOX shifted the band to around 58 kDa (Fig. S1), giving about 29% *N*-glycosylation of *Tv*GLOX which is higher than ~ 18% *N*-glycosylation of GLOX from *P. chrysosporium,* heterologously expressed in *P. pastoris*^26^.

### Biochemical characterization of *Tv*GLOX

*Tv*GLOX showed the highest activity towards methylglyoxal at pH 6.5 (Fig. S3) which is similar to other GLOX enzymes^1,3,21,23^ and retained about 100% of its initial activity after 5 h incubation at 25 °C and pH 6.5 (Fig. S4). After 3 h incubation of *Tv*GLOX between 40 °C and 60 °C, about 90% of its initial activity remained, whereas at 70 °C, it lost its activity immediately (Fig. S4). The thermostability of other GLOX enzymes like *Pci*GLOX1 and *Pci*GLOX2 at 40 °C and 50 °C was comparable to that of *Tv*GLOX, while at 60 °C *Pci*GLOX1 and *Pci*GLOX2 retained only 50% of their initial activity after 2 h of incubation^23^.

**Figure 3:**
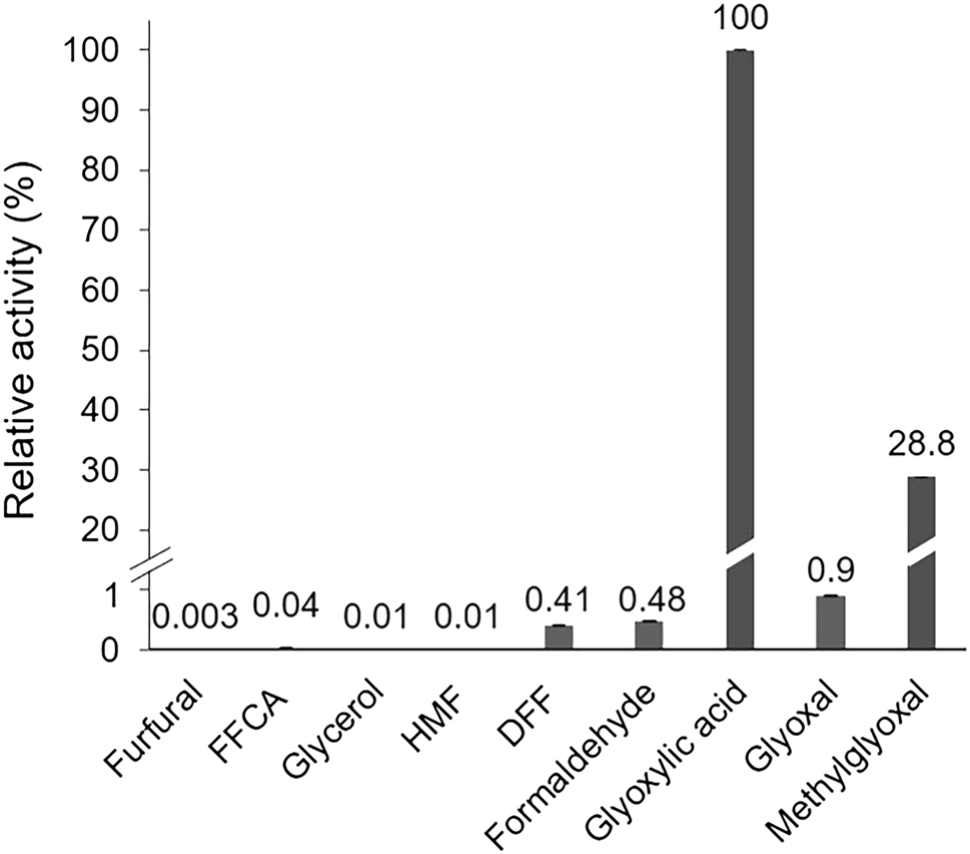
Substrate spectrum of *Tv*GLOX (activity towards glyoxylic acid was set to 100%).

**Figure 4:**
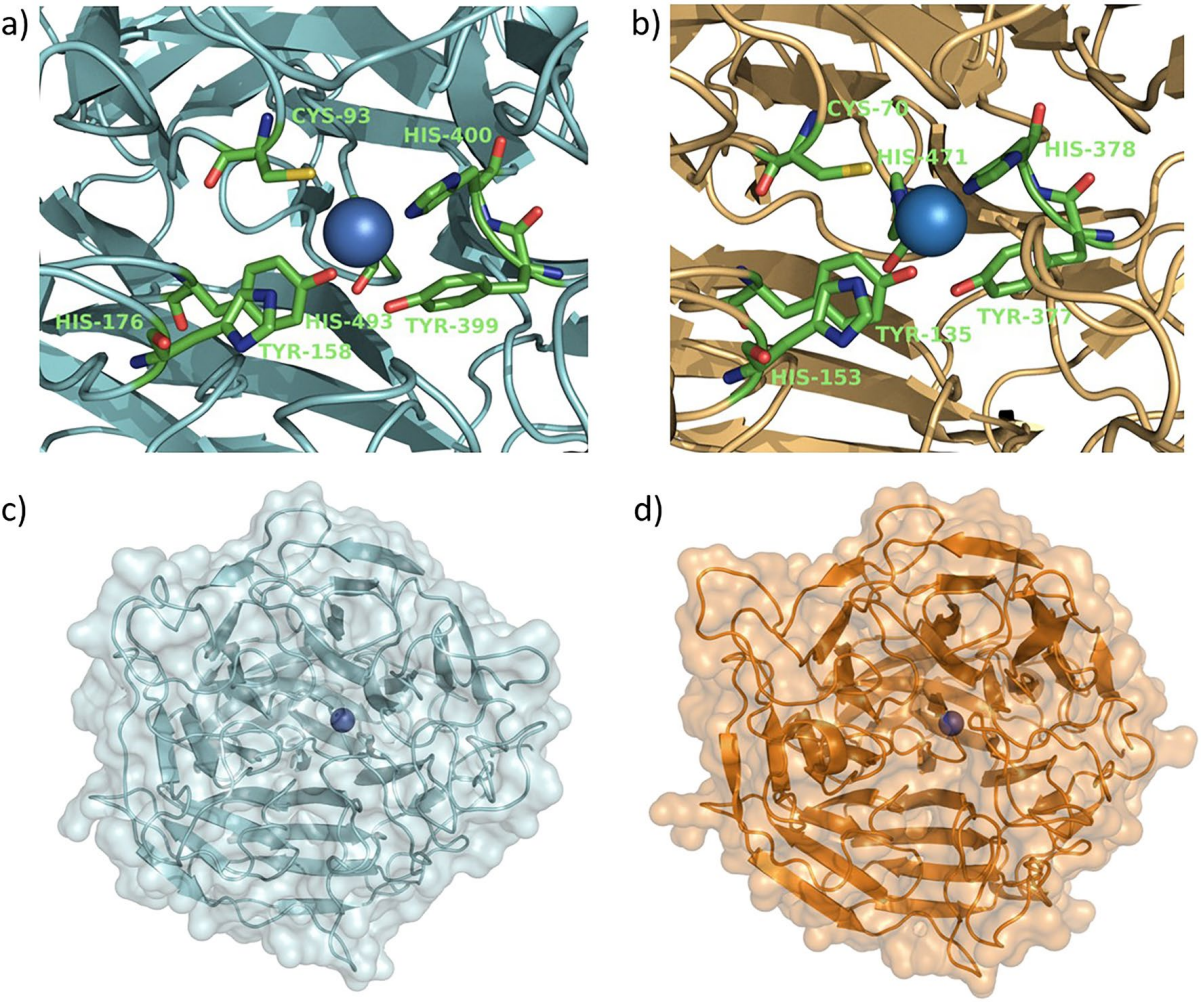
Structural model of *Tv*GLOX and GLOX from *P. chrysosporium*. Homology models of both enzymes were built using GalOx (PDB: 2EIE) as template. Catalytic site of (**a**) *Tv*GLOX and (**b**) GLOX from *P. chrysosporium*. The crosslink between the cysteine-tyrosine cofactor is missing due to the method of homology model building. Cartoon and surface representation of (**c**) *Tv*GLOX and (**d**) GLOX from *P. chrysosporium.* The copper ion is depicted as a blue sphere.

The substrate spectrum of *Tv*GLOX was estimated in a coupled assay, including HRP and ABTS, to measure H_2_O_2_ generated by *Tv*GLOX during substrate oxidation. A total of 15 compounds including HMF, HMFCA, DFF, FFCA, furfural, glycerol, formaldehyde, glyoxal, methylglyoxal, glyoxylic acid, D-galactose, D-glucose, benzyl alcohol, veratryl alcohol, and veratral were tested. As shown in Fig. 3, *Tv*GLOX possessed the highest activity towards glyoxylic acid followed by methylglyoxal, glyoxal, formaldehyde and DFF. Activity towards glycerol, HMF, FFCA and furfural was also detected, while no activity was found towards d-glucose, d-galactose, benzyl alcohol, veratryl alcohol, veratral and HMFCA. These results confirm that *Tv*GLOX is a typical glyoxal oxidase, that mainly accepts aldehyde groups and hardly oxidizes alcohol groups. In comparison, *Pci*GLOX1 also demonstrated the highest activity towards glyoxylic acid, whereas other GLOX enzymes such as *Pci*GLOX3 or GLOX from *P. chrysosporium* preferred methylglyoxal over glyoxylic acid^1,19,23^. Homology modeling confirmed that the active site and overall structure of *Tv*GLOX is quite similar to other GLOX enzymes such as GLOX from *P. chrysosporium* (Fig. 4). Like in other CROs, the active site of *Tv*GLOX contains an axial tyrosine (Tyr399), two histidines (His400 and His493), and a cross-linked cysteine-tyrosyl radical cofactor formed by Cys93 and Tyr158.

### Oxidation of HMF and derivatives thereof by *Tv*GLOX

Although GLOX typically oxidizes aldehydes to the corresponding acids and thus converts HMF to HMFCA, this enzyme is also capable of oxidizing alcohols to aldehydes, like glycerol to glyceraldehyde. While *Pci*GLOX1, *Pci*GLOX2, and *Pci*GLOX3 have been reported to predominantly oxidize the aldehyde group of HMF and to produce HMFCA^1^, *Mt*GLO*x* from *Myceliophthora thermophila* M77 has been shown to oxidize the alcohol group of HMF leading to DFF, but failed to further oxidize DFF^3^. We investigated the activity of *Tv*GLOX towards HMF, DFF, HMFCA, and FFCA in more detail and determined the corresponding product profiles in these reactions (Table 1). With HMF, *Tv*GLOX was mainly active on the aldehyde group and converted HMF to 88% HMFCA and about 12% FDCA after 24 h. Conversion of HMF by *Pci*GLOX2 and *Pci*GLOX3 reached only 39% and 41%, respectively after 24 h^1^. In contrast to *Mt*GLOX, which failed to oxidize DFF^3^, *Tv*GLOX showed the highest activity towards DFF among the investigated furan derivatives and completely converted DFF via FFCA to FDCA within 24 h.

**Table 1:** Conversion of furan derivatives catalyzed by *Tv*GLOX.

Detected compounds	Initial substrate								
	HMF		DFF			HMFCA		FFCA	
	2 h	24 h	2 h	24 h		2 h	24 h	2 h	24 h
	Product distribution (%)								
HMF	57.4	0.0	0.0	0.0		0.0	0.0	0.0	0.0
DFF	0.0	0.0	0.0	0.0		0.0	0.0	0.0	0.0
HMFCA	42.6	88.1	0.0	0.0		100.0	100.0	0.0	0.0
FFCA	0.0	0.0	20.9	0.0		0.0	0.0	14.7	0.0
FDCA	0.0	11.9	79.1	100.0		0.0	0.0	85.3	100.0

With HMFCA no product formation was detected after 24 h, which is consistent with the preference of *Tv*GLOX for the aldehyde group (as in HMF) and low acceptance of alcohol groups (as in HMFCA). According to this, the aldehyde group of FFCA was oxidized very well, leading to 100% FDCA after 24 h. As HMFCA was not accepted at all, the formation of small amounts of FDCA in the conversion of HMF with *Tv*GLOX might be explained by very slow oxidation of HMF to DFF (not observed after 2 h of reaction), followed by a fast conversion of DFF to FFCA and further to FDCA within 24 h of reaction. In contrast, with *Pci*GLOX1-3 only a low conversion of HMFCA to FFCA and FDCA within 24 h has been reported^1^. The absence of oxidation products in the reaction of *Tv*GLOX with HMFCA may also be related to stability issues caused by this furan derivative. Therefore, we investigated the influence of the furan derivatives on the stability of *Tv*GLOX. Only a slight decrease in activity was observed when *Tv*GLOX was incubated for 24 h with HMFCA or FFCA, while no loss in activity was observed with HMF and DFF (Fig. S5). Other GLOX enzymes are more sensitive to furan derivatives, e.g. all three GLOX enzymes from *P. cinnabarinus* almost completely lost their activity after 24 h incubation with HMFCA^1^. From our results, it can be concluded, that the lack of product formation in the reaction of *Tv*GLOX with HMFCA can be attributed to the inability of this enzyme to oxidize the alcohol group of HMFCA.

### Redox activators for *Tv*GLOX

As known from the literature, GLOX easily undergoes inactivation by reduction of the active free-radical Cu(II)-state to the catalytically inactive non-radical Cu(I)-state requiring reactivation in the course of substrate oxidation reaction^17,19,24^. High-redox potential inorganic oxidants like potassium octacyanomolybdate (K_3_Mo(CN)_8_), sodium hexachloroiridate (Na_2_IrCl_6_) or Mn^3+^ can reactivate GLOX^18,24^. In ligninolytic cultures, GLOX from *P. chrysosporium* was fully activated in the presence of lignin peroxidase combined with its substrate veratryl alcohol^19^. In this system, lignin peroxidase consumed H_2_O_2_ produced by GLOX. HRP can also be used for GLOX activation when applied together with a suitable HRP substrate such as ABTS or veratryl alcohol^18,27^. Although the activating effect has not been completely understood, it has been suggested that a single-electron oxidant generated by HRP or other peroxidases, is used to regenerate the oxidized radical form of GLOX^28^. Recently, this hypothesis has been supported by the finding that ABTS cation radicals, generated through the oxidation by HRP in HRP/GLOX-coupled reactions, were in fact the species responsible for GLOX activation during methylglyoxal oxidation (Fig. 2)^17^. In order to find the most suitable redox activator for *Tv*GLOX, we tested different HRP substrates—ABTS, 2,6-dimethoxyphenol, catechol, methyl syringate, syringaldazine, *p*-hydroquinone, guaiacol, veratryl alcohol and Mn^2+^, respectively—as redox activators of *Tv*GLOX during oxidation of FFCA (Table 2). Mn^2+^ is not a common HRP substrate, but Mn^3+^-oxalate complexes were previously shown to strongly activate GLOX when HRP was included in the reaction, due to the HRP-catalyzed oxidation of Mn^2+^ to Mn^3+^^24^.

**Table 2:** Formation of FDCA (%) from 2 mM FFCA by *Tv*GLOX in the presence of HRP and different redox activators after 24 h.

Entry	HRP	Redox activator	FDCA (%)
1	–	–	5
2	+	–	18
3	+	ABTS	100
4	+	Methyl syringate	94
5	+	Mn^2+^	52
6	+	Veratryl alcohol	25
7	+	Syringaldazine	17
8	+	2,6-Dimethoxyphenol	10
9	+	Guaiacol	6
10	+	*p*-Hydroquinone	4
11	+	Catechol	3

In the absence of both, HRP and a redox activator, a slight formation of FDCA by *Tv*GLOX was detected after 24 h, which may indicate that *Tv*GLOX was not completely inactive after purification. *Mt*GLO*x* from *M. thermophila* M77 was found to be fully active even without the addition of peroxidases or oxidizing agents^3^. Addition of commercial HRP Type II improved FDCA formation to a certain extent (18% FDCA with HRP vs. 5% FDCA w/o HRP). Whether *Tv*GLOX was activated by any compounds contained in the commercially available HRP preparation or activated by the enzyme itself, as it was reported for GLOX from *P. chryosporium*, which was moderately activated when incubated with lignin peroxidase^19^, remains elusive. The HRP/ABTS-system gave the best results with complete FFCA conversion within 24 h, followed by the HRP/methyl syringate system (94% FDCA). When FFCA conversion was analyzed after 2 h, it became apparent that HRP/ABTS overperformed HRP/methyl syringate in activating *Tv*GLOX (85% FDCA vs. 62% FDCA, respectively). HRP/Mn^2+^ was also able to activate *Tv*GLOX, but only half the FFCA conversion was achieved compared to the reaction with ABTS. Veratryl alcohol only moderately activated *Tv*GLOX as was also reported by Kersten et al.^27^, and resulted only in 25% conversion of FFCA. Syringaldazine did not improve FFCA conversion compared to the reaction without any redox activator, whereas 2,6-dimethoxyphenol, guaiacol, *p*-hydroquinone and catechol reduced FFCA conversion. Inhibition of GLOX by guaiacol and catechol when peroxidase was added to the reaction has been described before^27^. Consequently, the best HRP substrate, ABTS, with the strongest activating effect on *Tv*GLOX was used in further experiments.

### Reaction cascade for the production of FDCA from HMF

**Figure 5:**
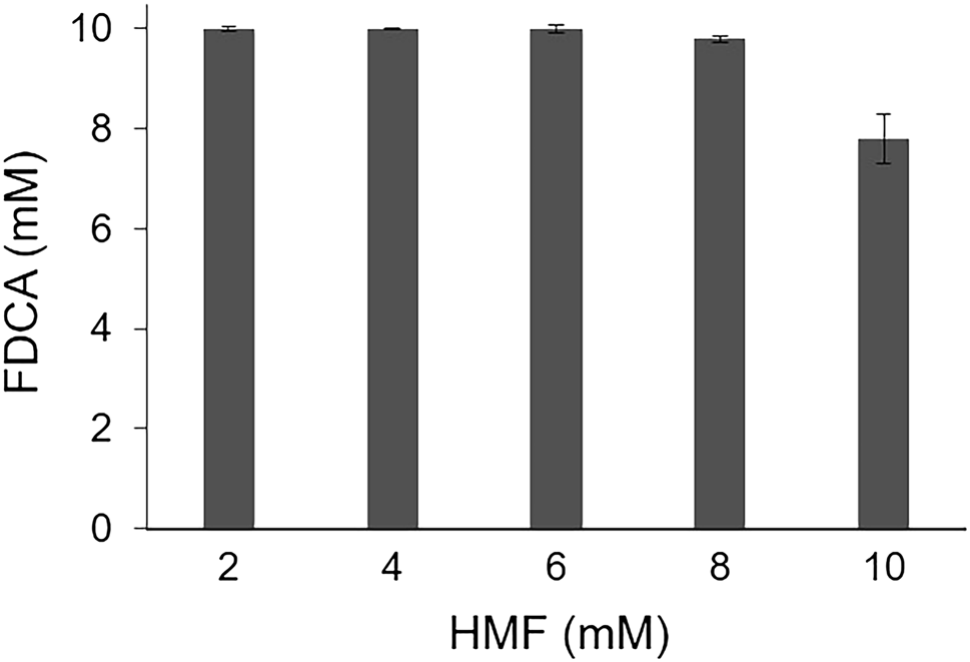
Dependence of FDCA production in the *Tv*GLOX/*Ma*AAO reaction system on increasing HMF concentrations.

In order to achieve the complete conversion of HMF to FDCA and avoid the accumulation of the dead-end product HMFCA, a cascade involving a second enzyme, the aryl-alcohol oxidase *Ma*AAO from *Moesziomyces antarcticus*, was established (Fig. S6). In our previous work, *Ma*AAO was found to convert HMF to FFCA within 24 h, but was not able to convert FFCA to FDCA^29^. We proposed that by combining the activities and substrate preferences of *Ma*AAO and *Tv*GLOX, complete conversion of HMF to FDCA can be achieved. Several experimental setups were tested. *Tv*GLOX was added after 2 h and 24 h of reaction with *Ma*AAO, respectively, and in one setup both enzymes were added from the beginning of the reaction. When *Tv*GLOX and *Ma*AAO were added at the same time as the substrate HMF, complete conversion of 2 mM HMF to FDCA was achieved after 24 h (Fig. 5). In a similar approach, Daou et al. combined *Um*AAO from *Ustilago maydis* and *Pci*GLOX3 from *P. cinnabarinus* in a cascade for HMF conversion to FDCA, but detected only 16% FDCA along with 84% FFCA^1^. In that study, catalase was also added to decompose high concentrations of H_2_O_2_ but conversion could be only marginally improved. Comparison with our results allows us to suggest that continuous reactivation of GLOX has a strong effect on the cascade outcome and GLOX inactivation by H_2_O_2_ only plays a marginal role.

In the next step, we increased the HMF concentration while maintaining the enzyme load, and observed complete conversion of up to 6 mM HMF to FDCA. With 8 mM HMF 7.8 mM FDCA and 0.2 mM FFCA were measured and with 10 mM HMF 7.8 mM FDCA and 2.2 mM FFCA (Figs. 5 and S7). For comparison, in a three-enzyme system consisting of a galactose oxidase, an unspecific peroxygenase, and an aryl-alcohol oxidase, 7.9 mM FDCA was achieved after 24 h starting from 9.7 mM HMF^7^. Our results show that *Tv*GLOX is a promising biocatalyst for the enzymatic synthesis of bioplastics precursors, and the continuous reactivation of this Cu-radical oxidase represents a critical factor during process optimization.

## Conclusion

In this study, a new glyoxal oxidase from *T. versicolor*—*Tv*GLOX—was heterologously expressed at high levels in *P. pastoris* and characterized regarding its substrate spectrum, stability and reactivation by different redox activators. *Tv*GLOX was most efficiently reactivated by ABTS in the presence of horseradish peroxidase (HRP) and preferred aldehydes over alcohols, leading to HMFCA formation from HMF. HMFCA was not accepted by this enzyme preventing further oxidation to yield FDCA. To achieve complete conversion to FDCA, a biocatalytic system consisting of *Ma*AAO and *Tv*GLOX, the latter one continuously reactivated through HRP and ABTS, was developed. With this system, up to 8 mM HMF was almost completely converted to FDCA within 24 h, demonstrating that *Tv*GLOX is a promising biocatalyst for biotechnological applications.

## Methods and material

### Construction of recombinant *Pichia pastoris* strain

*Tv*GLOX from *Trametes versicolor* FP-101664 SS1 (NCBI accession number XM_008037054) was identified by protein BLAST search using GLOX from *T. cinnabarina* (GenBank: ANJ20632.1) as query (88.91% identity, E-value 0.0, query coverage 100%). The *tvglox* encoding sequence with its native signal sequence for secretion was synthesized by BioCat GmbH (Heidelberg, Germany) after codon optimization for expression in *Saccharomyces cerevisiae* using the online tool JCat^25^. The gene was cloned into the *Pichia pastoris* expression vector pPICZA between EcoRI and NotI restriction sites (pPICZA_*tvglox*). Chemically competent *E. coli* DH5α cells were used for plasmid propagation. Transformants were selected on low salt lysogeny broth agar plates including 25 μg/ml Zeocin. Plasmid isolation was performed by using the ZR Plasmid Miniprep Kit (Zymo Research, USA). Electrocompetent *P. pastoris* X-33 cells were transformed by electroporation with 10 µg pPICZA_*tvglox* plasmid after linearization with MssI. Positive transformants were selected on yeast extract peptone dextrose sorbitol (YPDS) agar plates supplemented with 100 μg/ml Zeocin after incubation at 30 °C for 3 days.

### Heterologous expression of *Tv*GLOX in shaking flask and bioreactor

*P. pastoris* transformants with *Tv*GLOX activity were selected on buffered minimal methanol (BMM) screening agar plates (1.34% yeast nitrogen base, 100 mM potassium phosphate buffer pH 6.0, 4∙10^–5^ biotin, 0.5% methanol, 2% agar, 0.5 mM 2,2′-azino-di-(3-ethylbenzthiazoline sulfonic acid (ABTS), 0.006 mg/ml HRP, 2 mM methylglyoxal). Colonies with the strongest green halo formation after 72 h incubation at 30 °C were selected for cultivation in shaking flasks. For this purpose, a colony from the agar plate was grown in 10 ml BMGY (buffered complex glycerol) medium overnight at 30 °C and 200 rpm. The overnight-culture was used for the inoculation of 200 ml BMMY (buffered complex methanol) medium to an OD_600_ of 1 and incubated for 3–4 days at 25 °C and 200 rpm. Every 24 h, the cultures were fed with 0.5% (v/v) methanol. The OD_600_ value and volumetric activity of the supernatant were measured daily using methylglyoxal as substrate.

The most active *P. pastoris* transformant was used for fed-batch fermentation. Fed-batch fermentation was performed in a 7.5 L bioreactor (Infors, Switzerland) as previously described^30^. The temperature was set to 25 °C after induction of *Tv*LGOX expression and the fermentation proceeded for 9 days with daily sampling to follow OD_600_ and volumetric activity towards methylglyoxal.

### Enzyme purification

*P. pastoris* cells were separated from fermentation broth by centrifugation at 4 °C and 11,325×*g* for 15 min, and the collected supernatant containing secreted *Tv*GLOX was concentrated and rebuffered with 25 mM sodium phosphate pH 6.0 by tangential flow filtration (TFF, 10 kDa molecular cut-off; Pall, Port Washington, USA). *Tv*GLOX was purified via hydrophobic interaction chromatography (HIC) on an ÄKTA purifier FPLC system (GE Healthcare, USA). At first, 10 ml concentrated supernatant was incubated in 25 mM sodium phosphate buffer pH 6.0 containing 1.5 M ammonium sulfate at 10 °C overnight. Then, the sample was centrifuged at 4 °C and 18,000×*g* for 30 min, filtered and applied on the column with butyl sepharose HP medium. Elution was performed by decreasing ammonium sulfate concentration by adding 25 mM sodium phosphate buffer pH 6.0. Fractions with activity towards methylglyoxal were concentrated and desalted via an ultrafiltration membrane filter (Vivaspin Turbo 15, 10 kDa molecular cut-off). The purified *Tv*GLOX enzyme was kept at 4 °C until use.

### Biochemical characterization

The activity of *Tv*GLOX was routinely measured in 96 well microtiter plates in a coupled assay using horseradish peroxidase (HRP, Type II; purchased from Sigma-Aldrich (Schnelldorf, Germany)) and ABTS for initial activation of GLOX and monitoring of substrate oxidation through H_2_O_2_ formation. Reaction mixtures contained 20 μl *Tv*GLOX solution, 20 μl 100 mM methylglyoxal, 20 μl 0.06 mg/ml HRP, 20 μl 5 mM ABTS, and 120 μl 50 mM sodium phosphate buffer pH 6.5, including 0.5 μM H_2_O_2_. ABTS oxidation was followed at 420 nm using a Infinite M200 Pro plate reader (Tecan, Switzerland).

Protein concentration was measured using the Bradford method^31^. Deglycosylation of purified *Tv*GLOX (20 μg) was carried out under native and denaturing conditions (for up to 24 h) by using Peptide N-Glycosidase F (PNGase F, New England Biolabs, Germany) according to the manufacturer’s instructions. Purified and deglycosylated protein was visualized by SDS-PAGE^32^. Potential *N*-glycosylation sites were predicted by using the NetNGlyc 1.0 server prediction tool^33^.

pH optimum of purified *Tv*GLOX was measured in 40 mM Britton-Robinson buffer at several pH values between pH 2.0–11.5 by using methylglyoxal as substrate as described above. The stability of *Tv*GLOX at pH 6.5 was investigated by incubating the enzyme in 50 mM sodium phosphate buffer pH 6.5 at 25 °C for 5 h. Thermal stability was determined by incubating *Tv*GLOX in 50 mM sodium phosphate buffer pH 6.5 at different temperatures (40 °C, 50 °C, 60 °C, and 70 °C) for 3 h. After incubation, samples were taken, incubated on ice and the remaining activity was measured at room temperature using methylglyoxal as substrate.

The enzyme activity towards 15 compounds (HMF, HMFCA, DFF, FFCA, furfural, glycerol, formaldehyde, glyoxal, methylglyoxal, glyoxylic acid, d-galactose, d-glucose, benzyl alcohol, veratryl alcohol, and veratral) was estimated at a substrate concentration of 10 mM in a coupled assay as described above.

The enzyme stability towards furan derivatives was assessed by incubating *Tv*GLOX in 50 mM sodium phosphate buffer pH 6.5 with 2 mM substrate (HMF, DFF, HMFCA, and FFCA, respectively). Samples were taken after 4 h, 24 h, and 48 h incubation, and the remaining activity towards methylglyoxal was measured as described above.

### Oxidation of furan derivatives

Reactions were performed in a total volume of 200 µl in 50 mM sodium phosphate buffer pH 6.5 with 2 mM substrate (HMF, DFF, HMFCA and FFCA, respectively), 2 μM *Tv*GLOX, 0.5 μM H_2_O_2_, 0.1 mg/ml HRP and 0.5 mM ABTS. Reactions were incubated at 25 °C and 500 rpm for up to 24 h. Fifty μl samples were taken after 15 min, 2 h and 24 h, acidified with 10 µl 6 M HCl and 2-furoic acid (20 mM) was added as an internal standard. Methyl *tert*-butyl ether (MTBE) was used for sample extraction, each sample was dried over MgSO_4_, evaporated to dryness and resuspended in 50 µl of *N,O*-bis (trimethylsilyl) trifluoroacetamide (BSTFA), and incubated at 60 °C for 30 min before GC–MS analysis.

### Investigation of redox activators

Reactions were performed in a total volume of 200 µl in 50 mM sodium phosphate buffer pH 6.5 with 2 mM FFCA, 2 μM *Tv*GLOX, 0.5 μM H_2_O_2_, 0.1 mg/ml HRP and 2 mM redox activator (2,6-dimethoxyphenol, catechol, methyl syringate, syringaldazine, *p*-hydroquinone, guaiacol, veratryl alcohol and MnSO_4_, respectively). Reactions with HRP and Mn^2+^ were conducted in 50 mM sodium malonate buffer pH 6.5. Reactions were incubated at 25 °C and 500 rpm for up to 24 h. Fifty μl samples were taken in the course of the reaction and prepared for analysis via GC–MS measurements as described above.

### Optimization of HMF conversion

For optimization of the reaction cascade *Moesziomyces antarcticus* aryl alcohol oxidase (*Ma*AAO) was used^29^. Reactions were performed in a total volume of 200 µl in 50 mM sodium phosphate buffer pH 6.5 containing 2–10 mM HMF, 0.5 μM H_2_O_2_, 2 μM *Ma*AAO, 0.1 mg/ml HRP, and 0.5 mM ABTS. All reactions were incubated at 25 °C, 500 rpm for up to 24 h. Samples were taken in the course of the reaction and prepared for analysis via GC–MS measurements as described above.

### GC–MS analysis

Oxidation of furan derivatives was analyzed using a GC–MS-QP-2010 (Shimadzu, Japan) on an FS-Supreme-5 ms column (CS Chromatographie Service GmbH, Germany). The injection, interface, and ion source temperatures were set to 250 °C, 285 °C, and 200 °C, respectively. The temperature for the column was adjusted to 110 °C, maintained for 2 min, then increased to 300 °C gradually with 20 °C/min. Substrates and products were identified by comparing the measured mass spectra with authentic standards. Substrate conversion and product formation were calculated from substrate depletion (control set to 100%), using 2-furoic acid as the internal standard and from external calibration curves of furan derivatives (0–2 mM) and 2-furoic acid as internal standard.

### Supplementary Information

Supplementary Figures.

## Data Availability

The data that support the findings of this study are available from the corresponding author upon reasonable request.

## References

[1] Daou M (2019). *Pycnoporus*
*cinnabarinus* glyoxal oxidases display differential catalytic efficiencies on 5-hydroxymethylfurfural and its oxidized derivatives. Fungal. Biol. Biotechnol..

[2] Daou M, Faulds CB (2017). Glyoxal oxidases: Their nature and properties. World J. Microbiol. Biotechnol..

[3] Kadowaki MAS (2018). Characterization of a new glyoxal oxidase from the thermophilic fungus *Myceliophthora*
*thermophila* M77: Hydrogen peroxide production retained in 5-hydroxymethylfurfural oxidation. Catalysts.

[4] Agblevor FA, Jahromi H (2018). Aqueous-phase synthesis of hydrocarbons from furfural reactions with low-molecular-weight biomass oxygenates. Energy Fuel..

[5] Sayed M (2022). Oxidation of 5-hydroxymethylfurfural with a novel aryl alcohol oxidase from *Mycobacterium* sp. MS1601. Microb. Biotechnol..

[6] Vinambres M, Espada M, Martinez AT, Serrano A (2020). Screening and evaluation of new hydroxymethylfurfural oxidases for furandicarboxylic acid production. Appl. Environ. Microbiol..

[7] Karich A, Kleeberg SB, Ullrich R, Hofrichter M (2018). Enzymatic preparation of 2,5-furandicarboxylic acid (FDCA)-a substitute of terephthalic acid-by the joined action of three fungal enzymes. Microorganisms.

[8] Dijkman WP, Groothuis DE, Fraaije MW (2014). Enzyme-catalyzed oxidation of 5-hydroxymethylfurfural to furan-2,5-dicarboxylic acid. Angew. Chem. Int. Ed..

[9] Milic M, Byström E, de María PD, Kara S (2022). Enzymatic cascade for the synthesis of 2,5-furandicarboxylic acid in biphasic and microaqueous conditions: 'Media-agnostic' biocatalysts for biorefineries. ChemSusChem.

[10] Serrano A (2019). Complete oxidation of hydroxymethylfurfural to furandicarboxylic acid by aryl-alcohol oxidase. Biotechnol. Biofuels.

[11] Mathieu Y (2020). Discovery of a fungal copper radical oxidase with high catalytic efficiency toward 5-hydroxymethylfurfural and benzyl alcohols for bioprocessing. ACS Catal..

[12] Dijkman WP, Fraaije MW (2014). Discovery and characterization of a 5-hydroxymethylfurfural oxidase from *Methylovorus* sp. strain MP688. Appl. Environ. Microbiol..

[13] Carro J (2015). 5-hydroxymethylfurfural conversion by fungal aryl-alcohol oxidase and unspecific peroxygenase. FEBS J..

[14] Dashtban M, Schraft H, Syed TA, Qin W (2010). Fungal biodegradation and enzymatic modification of lignin. Int. J. Biochem. Mol. Biol..

[15] Whittaker MM, Kersten PJ, Cullen D, Whittaker JW (1999). Identification of catalytic residues in glyoxal oxidase by targeted mutagenesis. J. Biol .Chem..

[16] Whittaker JW (2002). Galactose oxidase. Adv. Protein Chem..

[17] Wohlschlager L, Kracher D, Scheiblbrandner S, Csarman F, Ludwig R (2021). Spectroelectrochemical investigation of the glyoxal oxidase activation mechanism. Bioelectrochemistry.

[18] Whittaker MM (1996). Glyoxal oxidase from *Phanerochaete*
*chrysosporium* is a new radical-copper oxidase. J. Biol. Chem..

[19] Kersten PJ (1990). Glyoxal oxidase of *Phanerochaete*
*chrysosporium*: Its characterization and activation by lignin peroxidase. Proc. Natl. Acad. Sci. USA..

[20] Kersten PJ, Kirk TK (1987). Involvement of a new enzyme, glyoxal oxidase, in extracellular H_2_O_2_ production by *Phanerochaete*
*chrysosporium*. J. Bacteriol..

[21] Wohlschlager L, Csarman F, Zrilic M, Seiboth B, Ludwig R (2021). Comparative characterization of glyoxal oxidase from *Phanerochaete*
*chrysosporium* expressed at high levels in *Pichia pastoris* and *Trichoderma*
*reesei*. Enzyme Microb. Technol..

[22] Leuthner B (2005). A H_2_O_2_-producing glyoxal oxidase is required for filamentous growth and pathogenicity in *Ustilago** maydis*. Mol. Genet. Genomics.

[23] Daou M, Piumi F, Cullen D, Record E, Faulds CB (2016). Heterologous production and characterization of two glyoxal oxidases from *Pycnoporus*
*cinnabarinus*. Appl. Environ. Microbiol..

[24] Roncal T, Munoz C, Lorenzo L, Maestro B, de Guerenu MDD (2012). Two-step oxidation of glycerol to glyceric acid catalyzed by the *Phanerochaete chrysosporium* glyoxal oxidase. Enzyme Microb. Technol..

[25] Grote A (2005). JCat: A novel tool to adapt codon usage of a target gene to its potential expression host. Nucleic Acids Res..

[26] Son YL, Kim HY, Thiyagarajan S, Xu JJ, Park SM (2012). Heterologous expression of *Phanerochaete*
*chrysoporium* glyoxal oxidase and its application for the coupled reaction with manganese peroxidase to decolorize malachite green. Mycobiology.

[27] Kurek B, Kersten PJ (1995). Physiological regulation of glyoxal oxidase from *Phanerochaete*
*chrysosporium* by peroxidase systems. Enzyme Microb. Technol..

[28] Pedersen AT (2015). Process requirements of galactose oxidase catalyzed oxidation of alcohols. Org. Process Res. Dev..

[29] Lappe A, Jankowski N, Albrecht A, Koschorreck K (2021). Characterization of a thermotolerant aryl-alcohol oxidase from *Moesziomyces antarcticus* oxidizing 5-hydroxymethyl-2-furancarboxylic acid. Appl. Microbiol. Biotechnol..

[30] Jankowski N, Koschorreck K, Urlacher VB (2020). High-level expression of aryl-alcohol oxidase 2 from *Pleurotus **eryngii* in *Pichia pastoris* for production of fragrances and bioactive precursors. Appl. Microbiol. Biotechnol..

[31] Bradford MM (1976). A rapid and sensitive method for the quantitation of microgram quantities of protein utilizing the principle of protein-dye binding. Anal. Biochem..

[32] Laemmli UK (1970). Cleavage of structural proteins during assembly of head of bacteriophage-T4. Nature.

[33] Kukuruzinska MA, Bergh MLE, Jackson BJ (1987). Protein glycosylation in yeast. Annu. Rev. Biochem..

