# Supplementary material for: Identification of redox activators for continuous reactivation of glyoxal oxidase from Trametes versicolor in a two-enzyme reaction cascade
Source: Sci Rep. 2024 Mar 11;14:5932. doi: 10.1038/s41598-024-56429-z (PMC10928124; doi:10.1038/s41598-024-56429-z)
Supplement: Supplementary file 1 — Supplementary Figures. [file 41598_2024_56429_MOESM1_ESM.pdf]

Identification of redox activators for continuous reactivation of glyoxal oxidase from *Trametes versicolor* in a two-enzyme reaction cascade

Saadet Alpdağtaş [1,2], Nina Jankowski [2], Vlada B. Urlacher [2], Katja Koschorreck [2,*]

[1] Department of Biology, Van Yuzuncu Yil University, Van 65080, Turkey

[2] Institute of Biochemistry, Heinrich Heine University Düsseldorf, Universitätsstraße 1, Düsseldorf 40225, Germany

* Corresponding author: Katja Koschorreck

e-mail-address: Katja.Koschorreck@hhu.de

phone number: +49 211 81 10749

Supplementary Information

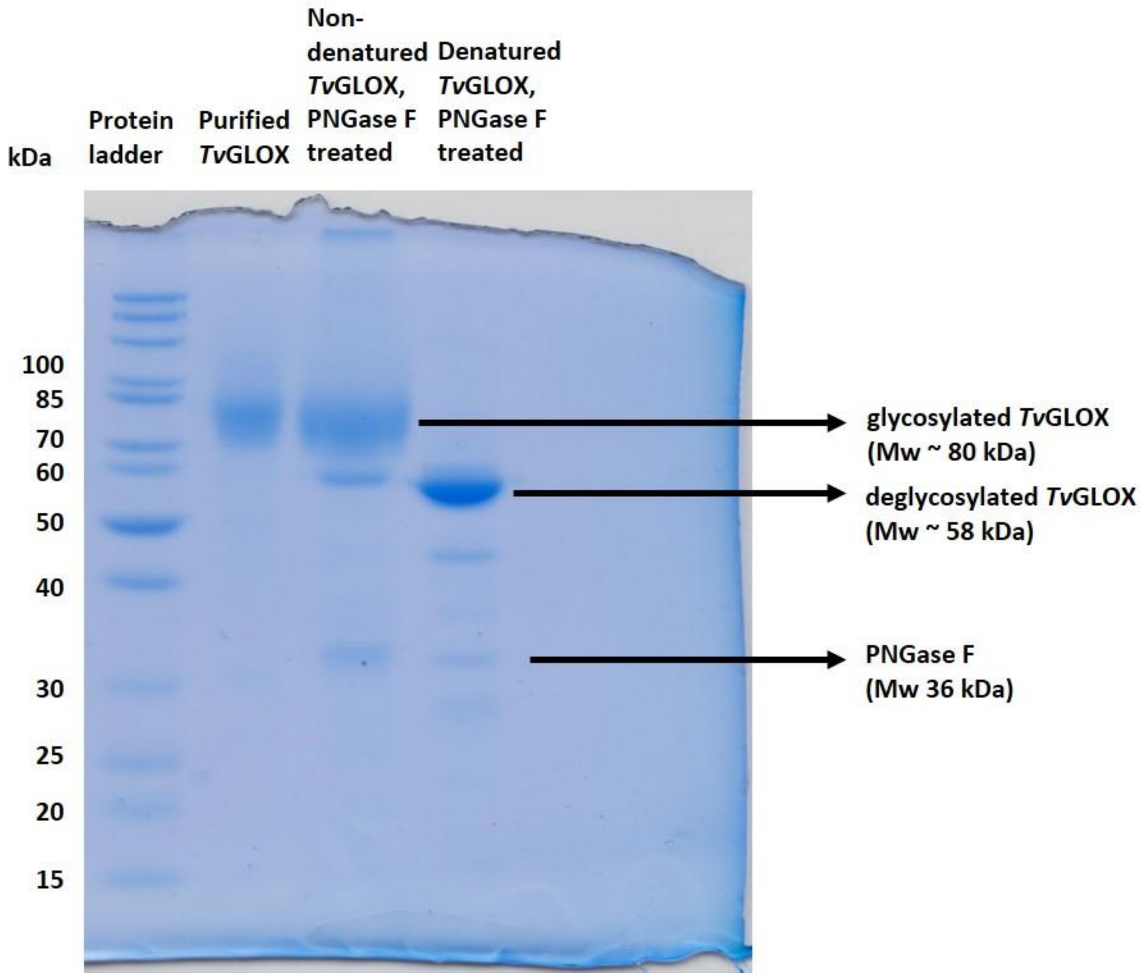

Figure S1. SDS-PAGE analysis of purified and PNGase F treated *Tv*GLOX.

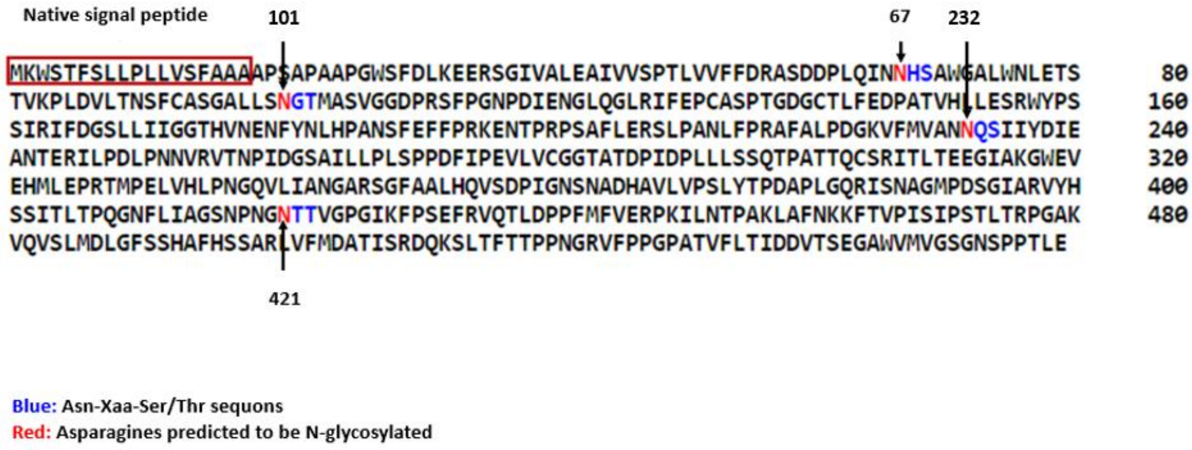

Figure S2. Predicted signal peptide and *N*-glycosylation sites of *Tv*GLOX. Numbered asparagine residues in red indicate possible *N*-glycosylation sites of *Tv*GLOX (sequons N-X-T/S, where X indicates any residue except for proline).

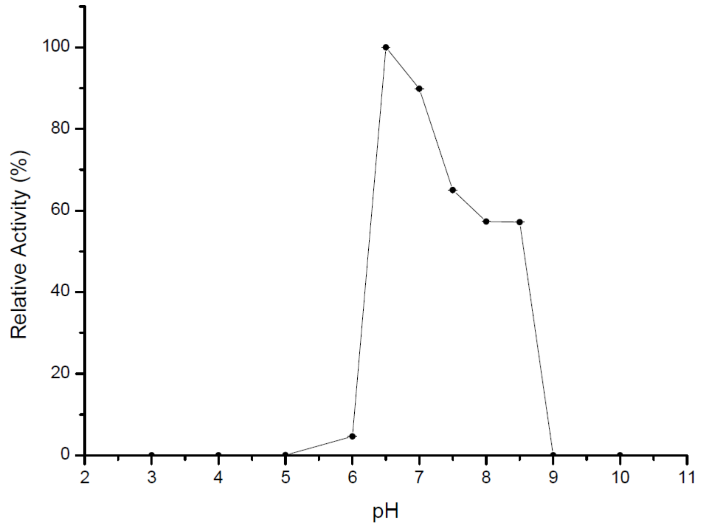

Figure S3. pH optimum of *Tv*GLOX towards methylglyoxal.

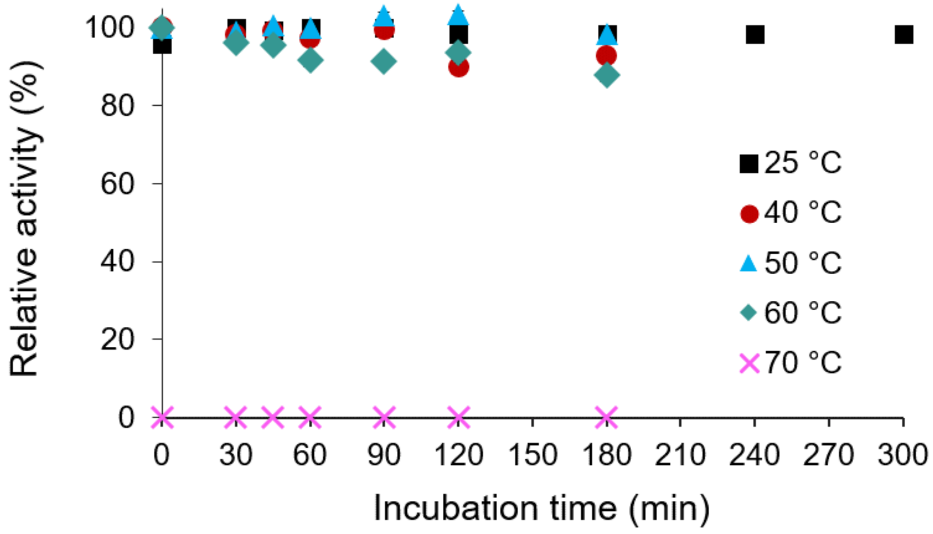

Figure S4. Stability of *Tv*GLOX at 25 °C to 70 °C at pH 6.5.

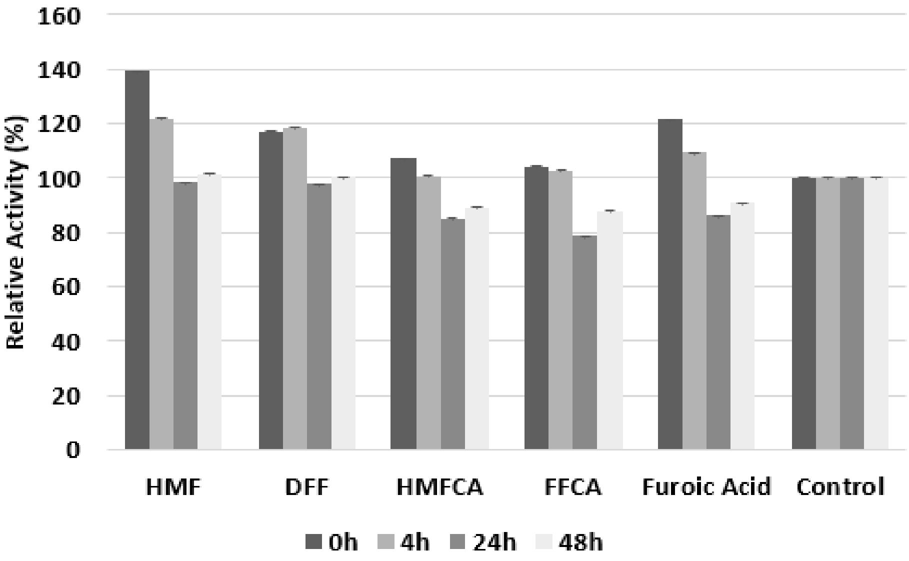

Figure S5. Stability of *Tv*GLOX in the presence of furan derivatives. *Tv*GLOX was incubated in 50 mM sodium phosphate buffer pH 6.5 with 2 mM of the respective furan derivative for up to 48 h. The activity was measured using methylglyoxal as substrate.

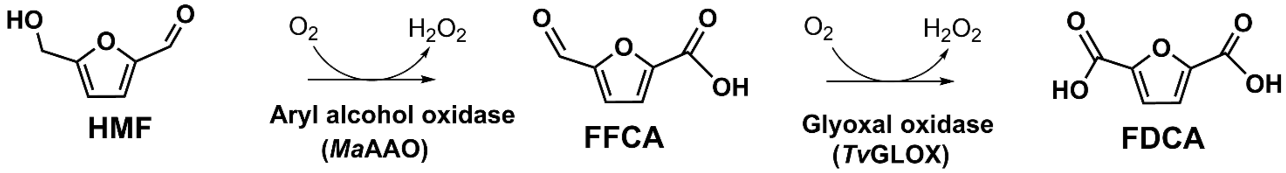

Figure S6. Two-enzyme system for FDCA production from HMF.

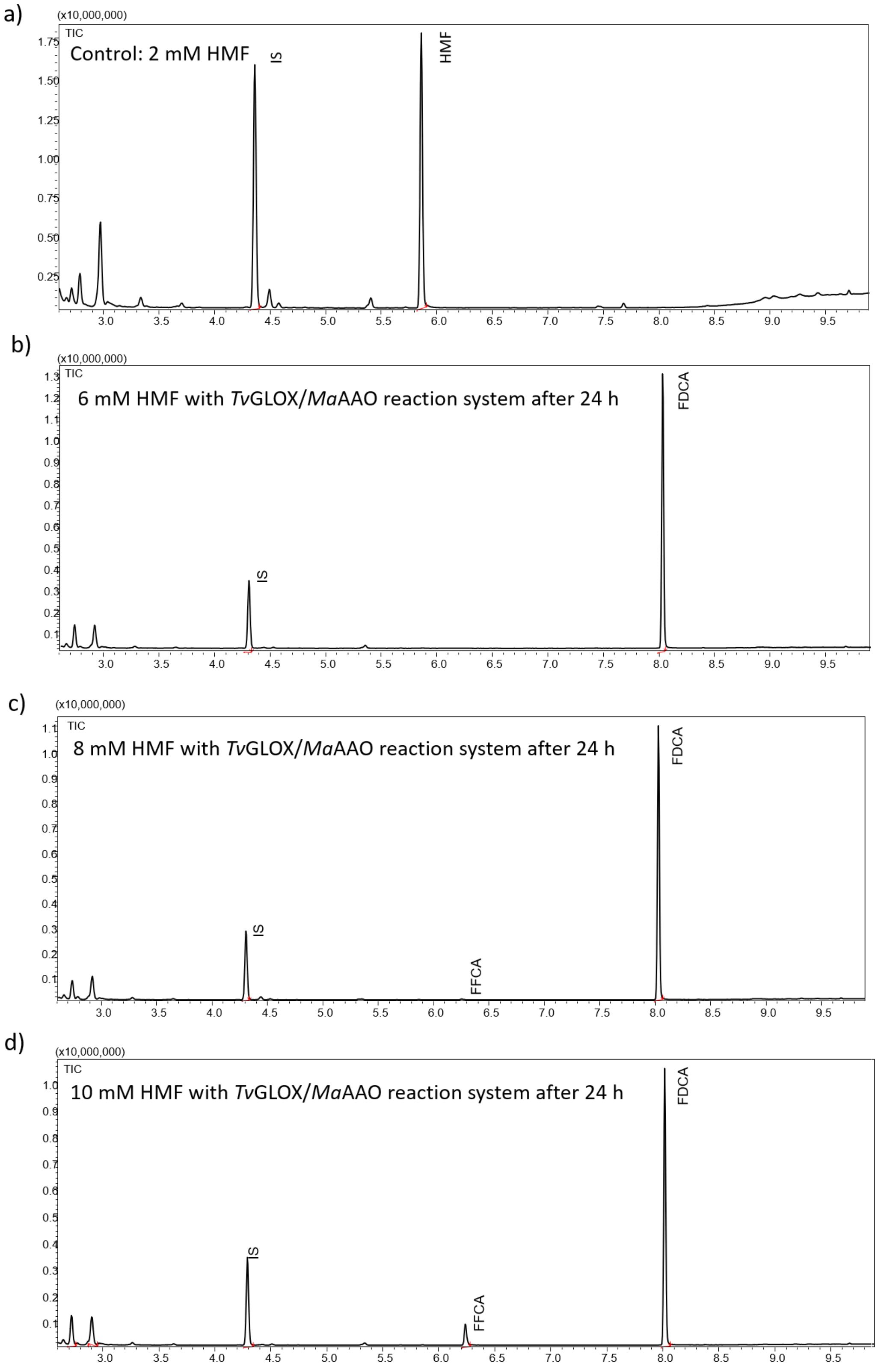

Figure S7. Conversion of HMF at different concentrations with the *Tv*GLOX/*Ma*AAO reaction system after 24 h. a) HMF control; b) 6 mM HMF; c) 8 mM HMF; d) 10 mM HMF.
